# Health Inequality as a Large-Scale Outcome of Complex Social Systems: Lessons for Action on the Sustainable Development Goals

**DOI:** 10.3390/ijerph17082684

**Published:** 2020-04-14

**Authors:** Anna Matheson

**Affiliations:** 1 School of Health, Te Herenga Waka, Victoria University of Wellington, Wellington 6140, New Zealand; anna.matheson@vuw.ac.nz; 2 Te Pūnaha Matatini, Centre for Complex Systems, Auckland 1142, New Zealand

**Keywords:** health inequality, complex social systems, complexity theory, social intervention, power relationships, evidence, social innovation, sustainable development goals

## Abstract

Action on the Sustainable Development Goals (SDGs) needs to become real and impactful, taking a “whole systems” perspective on levers for systems change. This article reviews what we have learned over the past century about the large-scale outcome of health inequality, and what we know about the behaviour of complex social systems. This combined knowledge provides lessons on the nature of inequality and what effective action on our big goals, like the SDGs, might look like. It argues that economic theories and positivist social theories which have dominated the last 150 years have largely excluded the nature of human connections to each other, and the environment. This exclusion of intimacy has legitimatised arguments that only value-free economic processes matter for macro human systems, and only abstract measurement constitutes valuable social science. Theories of complex systems provide an alternative perspective. One where health inequality is viewed as emergent, and causes are systemic and compounding. Action therefore needs to be intensely local, with power relationships key to transformation. This requires conscious and difficult intervention on the intolerable accumulation of resources; improved reciprocity between social groups; and reversal of system flows, which at present ebb away from the local and those already disadvantaged.

## 1. Introduction

Understanding how human driven systems behave needs to become more sophisticated. We need ways to comprehend human systems that can see past social identities and relations. Theories of the behaviour of complex systems provide a way forward. They provide a separation between complex processes—such as emergence, feedbacks and sensitivity to initial conditions—and the qualitative nature of humans and human society [1]. This does not make people and culture irrelevant to social progress and change (as dominant theories of economics and some strains of the social sciences have professed). Rather, it makes human subjectivity and connection more integral to acting effectively on emergent large-scale health, social and environmental outcomes. The last 30 years has seen a shift occurring towards more sophisticated conceptualisations of health intervention with those grounded in complex systems now gaining legitimacy. This article argues that theories of complex systems provide a way to understand *how* to achieve change, rather than only describing it [2].

In Aotearoa New Zealand (NZ) the systemic pathways that lead to health inequality are becoming more widely understood [3]. Whilst failure to act effectively on large-scale health outcomes internationally has led to calls for a paradigm shift away from linear, siloed approaches to health and social intervention [4,5,6,7]. This paper explores two areas of knowledge—the evidence of health inequalities, and what we know about intervening in complex systems. The last two decades have seen the volume of literature and analysis on these subjects increase substantially. The health inequalities literature has become compelling in the evidence it provides of the way social systems produce health outcomes—clearly demonstrating systemic and compounding social processes. Whilst in parallel there has been a significant shift to both normalising complex systems thinking about social challenges, and applying this thinking to social intervention [7,8]. Given we are seeing many aspects of human activity accelerating [9], it seems timely to reflect on what we know about how to intervene effectively.

This review draws on my experiences over, at least, the last decade where I have contributed to, and observed the evolution of research and thinking in these areas. To unpack and make sense of developments, this article first outlines some important conceptual framings for large-scale social intervention; second, I summarise what we know about the cumulative evidence of health inequalities; third, I introduce some key literature and applications of complex systems thinking; and finally, I discuss what this means for intervening in complex social systems reflecting on transferable lessons for the Sustainable Development Goals (SDGs) [10]—given their cross-over and comparability to the large-scale goal of reducing health inequalities.

## 2. Social Intervention: A Key Activity for Societies

Reducing inequality has frequently been a key intent of large-scale social structuring. From the grand ideals of communism, to “trickle-down” arguments within economic theory, to “grassroots” and “bottom-up” methods embraced by development practitioners—social intervention is a central activity and focus of societies. In 1998 James Scott described the “blindness” of pre-modern times in relation to the state’s knowledge of characteristics of its populations. He argued that much early modern European activity of the state was rationalising and standardising features of society into administratively convenient forms. Some of these early ways of increasing the “legibility” of society were the creation of surnames, recognising an official language and centralising traffic patterns [11].

Over the past few decades this legibility has increased exponentially with science and research on health, social, and environmental outcomes becoming more nuanced. At the same time, big data sources are more accessible and usable with vastly improved transmission enabled by the internet and analytical developments such as machine learning. This increased legibility has far-reaching consequences for the relationship between governments and populations, and state and non-state actors. Although better data and evidence to underpin policy decision-making makes possible discriminating interventions in areas such as public health and welfare - challenges have also arisen. Industries that are harmful to population health have been able to improve their marketing and targeting of products, as well as shift their production to environments with little government regulation. Furthermore, the acceleration in the spread of misinformation and ideologies has tended to increase social polarisation with consequences for political outcomes [12]. Even well-established democracies have become prone to this spread which exploits relationships between social groups.

The seduction of free-market capitalism (which gained renewed power in reaction to the collapse of Soviet collectivisation) was the belief that economic growth would expand the whole economy. Everyone’s wealth would ultimately increase, even if a degree of inequality remained. But this is not what we have seen. Rather than any reasonable, tolerable level of inequality there has been immense and increasing wealth inequality exacerbated by the interaction of population growth and technology. Oxfam, for example, recently reported that 22 men currently own greater wealth than all of the women in Africa [13]. We also now know that capitalism, in its current unfettered form, threatens the ability of the planet to sustain human life through climate and environmental exploitation and degradation. Even where there is political will, advanced democracies are struggling to intervene to regulate and hold to account organisations, corporations and individuals who have accumulated large amounts of wealth and power. The activities of these entities frequently span numerous countries with negative health, social, and environmental impacts on local communities [14].

Those who have benefitted most from the current version of capitalism, have ignored what has been intuitively known and practiced by many indigenous cultures throughout history and the world: That humans exist within the complex processes of systems that intimately tie us both to the Earth, and to the rest of life on Earth. Economic theories and positivist social theories which have dominated the last 150 years have largely excluded both the need for, and nature of human connections to each other and to the environment. This exclusion of intimacy within our theoretical and conceptual understandings has legitimised arguments that only value-free economic processes matter for macro human systems, and only abstract measurement constitutes valuable social science. As a result, we have been left with a dearth of theory about large-scale human processes, at a time when we urgently need effective approaches to intervention.

## 3. The Reproduction of Health and Social Inequality

The most enduring macro pattern human societies produce and reproduce is social inequality. It shapes the distribution of many large-scale outcomes—including infectious disease epidemics, obesity, heart disease, cancers, and environmental impacts. It influences causal pathways, through systematically advantaging and disadvantaging social groups. The volume of evidence and thinking about how social inequality impacts health outcomes is significant. The measurement and description of health inequality has a deep history stretching back to the mid-1800s when Frederick Engels wrote of the poor health, and short lives, of the working class in Britain [15]. Subsequently health inequality has been described in detail in a number of western countries [16,17,18,19,20,21,22]. Absolute inequality has been shown to exist *between* countries—for example average life expectancy in sub-Saharan Africa is 20 years less than many western countries [23]. We also know that relative inequality matters for health, with big differences in life expectancy between social groups and communities being observed *within* countries.

We have known for a long time that health inequality follows a pattern: Any measure of social circumstance—class, income, occupation, education—will show a gradient in health outcomes, with the advantaged having better health, and the less advantaged poorer health [24,25,26,27]. We also know that particular social groups defined by gender, ethnicity, religion or minority status, such as disability, new migrants and refugees, are impacted by health inequalities. The WHO Commission on the Social Determinants of Health in 2008 added to the weight of evidence arguing that “social injustice is killing people on a grand scale” [28]. The findings of the Commission unequivocally showed the unfair distribution globally of the social, commercial and political determinants of health.

We also know a lot about how inequality “gets under the skin”. Recent evidence has reconfirmed how disadvantage can be observed at multiple biological levels within individuals [29]. Adding to the ample social epidemiological evidence backing up the ground-breaking Whitehall study, showing how a lack of control in aspects of people’s lives leads to an increase in stress, which has biological consequences [30]. The evidence of multi-morbidities, where individuals will have a number of illnesses simultaneously, also shows how illness compounds [31].

There is plenty of evidence illustrating the importance to health of “social capital” in the form of what can be called “horizontal” social relationships, such as social support [32]. Whereas “vertical” social relationships are argued to be even more instrumental for health inequality - those relationships that can influence the ability of individuals and communities to act on economic, political or policy concerns [33]. Research continues to illuminate poor local health impacts resulting from the behaviour of industries, such as tobacco and alcohol, when they are left to pursue profit without state regulation and enforcement [34]. Furthermore evidence is accumulating that international trading arrangements and investment policies frequently favour developed economies and powerful transnational corporations with detrimental consequences for already poor communities [35].

The systemic nature of health inequalities is further explained by the substantial evidence of institutional racism and discrimination [36]. This is where health and social outcomes for particular social groups are shaped by prejudicial interactions occurring throughout the “whole system”. For example, health, welfare, criminal justice and economic systems in many western countries show poor outcomes for specific groups. In NZ, indigenous Māori women make up 60% and men more than 50% of the prison population, despite being 15% of the country’s total population. There is also clear evidence that Māori are systematically disadvantaged in terms of access to, and the journey through, the health system [37]. The same picture emerges from most of our economic and policy systems [38]. Consequently, for almost all health outcomes and their determinants, Māori fare less well than the general population, with an overall life expectancy seven years less than the NZ/European population [39]. Many have identified this phenomenon as institutional racism. Where rather than an explicit intent to produce inequality, it is the accumulation of interactions, many unintentional, that disadvantage particular groups and lead to measurably poorer outcomes [40].

Local geographic area has proven singularly important in the reproduction of health inequalities. Local areas connect people to each other, and to their histories, and to social, physical and service environments—those places where people spend most of their time and are most connected. This is where the results of systemic disadvantaging frequently plays out, compounding health outcomes within individuals and within communities [41,42]. This challenge of complexity has led to frameworks that incorporate local context, like socio-ecological and political economy perspectives [43].

For all this evidence and thinking, the transfer of knowledge—from understanding about population-level social patterns to informing action and intervention—has been slow. Even high-income countries (with very good evidence) are still grappling with how to effectively intervene to reduce health inequality. In response to the seemingly intractable nature of inequality, recent years have seen burgeoning literature internationally, arguing the relevance of complex systems thinking [7,44,45,46].

## 4. Real Complex Systems and the Theory of How They Behave

The biological, institutional, social and geographic evidence show how multiple levels of influence create health inequality. This observation of “levels” has led to contemporary social research methods, such as multi-level modelling, which quantify the causal influence of variables at different social levels. Importantly this type of approach recognises the layered character of causality but is nonetheless still derivative of a general linear model, unable to handle the complexity of reality [47,48].

In 2007 sociologist Sylvia Walby wrote of a hiatus in the development of thinking about large scale social processes, especially regarding systems, during the post-modern turn [49]. Walby described the era of post-modernism as a reaction against the meta-narratives of modernism. This reaction involving a trend within sociology to focus almost exclusively on ontological depth rather than more general theories of explanation. Walby argues that complexity theory provides a way past this polarising of methodology and that “ontological depth”—such as knowledge of sets of social relations in eco-social context—does not have to be at the expense of explanatory power [50]. The growing sophistication and popularity of intersectional methodologies highlights the traction in this area [51].

Developed during the 1980s, but new as coherent theories, complexity theory and its predecessor chaos theory, were the result of more than a century of scientific experimentation and theorising about how matter organises and behaves. Since the 1990s complexity theory began increasingly influencing thinking in a range of social sciences, including the health sciences where its use has become more widely accepted [5,6,7]. Complexity theory sees social phenomena, such as inequality, as the “emergent” result of interacting elements within a social system [52]. Emergent social phenomena are real in that they have an impact on people, the systems that generated them, and wider interacting systems [53]. Social systems evolve and behave like any other system, be it chemical, biological or physical. But social systems are, observably, objectively subjective. Uniquely, we humans have considerable consciousness where we can observe and act on systems at different scales. Through our innate abilities (including empathy and intellect), and through the tools and schemas we have created (such as science and research), we are not only able to observe our physical environment, but also our own social context. The increasing legibility of society has opened the opportunity to more effectively act with consciousness on the trajectories of social systems.

One of the few identifiable women leaders within the complexity sciences is Donella Meadows, a pioneering chemist and environmentalist. Meadows recognised *real* systems and developed ways to think about change from that perspective. In 1972 she led a seminal report which modelled the unsustainability of the trajectory of perpetual economic growth for Earth’s systems [54]. Through her knowledge of chemical systems Meadows developed a framework of what works to change and transform systems—any system [55]. She identified a hierarchy of levers of change and their effectiveness. These levers include changing information flows, feedback loops, system rules and, at the top of the hierarchy of effectiveness, creating paradigm shifts. These are the deep mechanisms or beliefs on which a system operates. Donella died suddenly in 2001. But her work on system behaviour has gained renewed currency, recently being applied to environmental degradation, the social determinants of health and climate change [56,57,58]. All these recent analyses conclude the need for better understanding of large-scale systems change and its multi-level levers.

## 5. Discussion: Social Intervention in Complex Social Systems

Already we know that social intervention has often led to an increase in social inequality, the opposite of what in most cases was intended. Unintended consequences of social intervention are not confined to high-level state social structuring. Interventions such as vaccination, health social marketing, technological advances, community initiatives and the health system itself, have been shown to reproduce and even increase inequality without conscious attention given to avoid this outcome [59,60]. In NZ for example we have had many years of health policies aimed at reducing health inequality for the Māori population. However, one recent report which backs up findings of several other high-level reviews of health, welfare and education in NZ, has shown despite intention and huge investment over 30 years, Māori inequality remains almost unchanged [61].

A key concept of complexity theory is that the whole is greater than the sum of the parts. Implying solutions cannot be piecemeal; they need to be informed by evidence about the “whole system”. The multi-level and locally compounding nature of the causes of health inequality need to be factored in. How artefacts, such as money and information and evidence flow through social systems and reach people, is not straightforward. “Trickle-down” economics for example says that if the wealthy have more resources, this increases their productivity, and results in them acquiring further wealth that will eventually reach the whole population. In practice we have found this is not what happens. Instead, policies and other actions implemented to foster this approach to growth—such as generous tax breaks for high-income earners—have protected and even accelerated incumbent system trajectories for some social groups, through exploiting the natural behaviour and flow of systems. This has led to increasing income inequality as a result of sensitivity to initial conditions; and has kept marginalised groups and communities excluded from the economic system, as a result of the lack of attention given to system boundaries and interactions. Instead interactions are amplified or stymied at the intersection of boundaries between “community” systems that are nested within each other and delineated by purpose, geography, beliefs and organisational, social and cultural norms. These social system “stocks and flows” have consequences for how money, ideas, information, evidence and practices reach and impact different communities.

Geographically based community interventions are, for good reason, often seen as an effective intervention approach. However, the evidence shows we have not seen the successes from them, we would have expected. There have been numerous community initiatives globally aimed at health and equity. Among those which have provided insight, are the Health Action Zones in the UK [62,63], the Healthy Cities initiative in a number of European countries [64], the Healthy Islands initiatives within the Western Pacific [65], the New Deal and Big Local community empowerment interventions in the UK [66,67], and the Steps to a Healthier US programme [68]. We have learned from these, and others, that when large-scale community interventions are planned and implemented without considering complexity, they suffer challenges to their potential effectiveness. Evaluating and attributing specific impacts can be difficult—especially in the short-term—because they usually span different community contexts, and take different forms [69]. More fundamentally, wider institutional and multi-level factors are frequently ignored. That is, even when local context is considered, vertical relationships are commonly left unaddressed, especially those related to power and resources. Reflections from the Health Action Zones, for example, show that the failures of the initiative lay in the nature of the policy system and its responses, rather than the communities themselves [62]. Because of challenges in showing efficacy, and their potential impact on existing power relationships, these types of initiatives are also very vulnerable to political change.

A challenge for community approaches to inequality is that real devolution rarely occurs. There appears to be a pattern whereby real power, resources and information are rarely returned to the local in any meaningful way. The Ottawa Charter for Health Promotion [70] goal of reorienting health services is a prime example of this, where evidence shows reorientation to a community and prevention focus has not been achieved adequately anywhere in the world [71]. Similarly in NZ, an attempt to reorient the health system through a strengthened primary care sector has so far not worked as planned [72]. Instead, existing power relationships between policy, professionals and communities have replicated themselves through the new organisational structures aimed at devolution.

Another woman of note, Penelope Hawe, has led thinking on public health and intervention arguing it is “an event within systems” [8]. That is, planned interventions need to recognise system context and be able to adapt to the specific social, economic, cultural and geographic circumstances of a community. In NZ, one such intervention is “Whānau Ora” which was borne from the Māori concept of family wellbeing [73] and connects up and organises health and social services around the needs of individuals, extended families and communities. This understanding of family – or whānau—sits within a much more relational worldview which is embedded in Māori culture. At the core of the intervention are Māori cultural values, but it relies on the power of central government decision-making being devolved to foster greater local control [74]. Another initiative, Healthy Families NZ, has come on the back of a recent surge in systems thinking for social action [75,76]. It explicitly aims to improve health and wellbeing in NZ through systems change. Currently run in nine communities, it uses a locally adaptive approach to make local environments healthier by targeting local and national leadership, policy and improving collective action. The approach to system change is intensely local, with local needs, cooperation, voice and methods being prioritised [77]. To date, and despite the disproportionate political and media scrutiny community-led initiatives are subject to, these two initiatives have so far weathered political change.

Social systems are hierarchical only in the sense that humans subjectively value some systems over others. At present the “local” is undervalued—from local workforces to local ecosystems. Largely, information, resources and human agency flow away from the local. It is therefore not just that disadvantaged communities require empowering, it is that the “local” in general needs empowering. Community health interventions hold promise for reversing inequality, but only if they can tackle the more difficult relationships between social groups. Those between social classes and different ethnicities, and between communities, governments and policy makers. Policy decisions are not made from a neutral position—they form an influential part of the community systems they are intervening in. The common thread from insights into community action lies in how we understand relationships of power. The population health and human behaviour disciplines are increasingly identifying the central, and causal role of power relationships [78,79]. Both perspectives see it as real, pivotal, and where the potential for large-scale social transformation lies.

### Lessons for the Sustainable Development Goals (SDGs)

Action on the 17 SDGs can take some lessons from this knowledge. First, inequality itself cannot be separate from other goals as it is integral to the “whole system” through its deeply relative and relational nature. Second, levers of change need to recognise the undervaluing of the local and pay attention to the way that power relationships limit real action and real reversals in the flows of information, resources and agency.

The intentions behind the SDGs are widely viewed as holding promise and an improvement on past approaches through their more explicit attention to inequality, and to implementation [80]. For example, Goal 3 targets health and well-being, Goals 5 and 10 explicitly target inequality, and within Goal 10 there are separate targets distinguishing within-country and between-country inequalities. There is also a separate goal (Goal 17) targeting the “means of implementation” with sub-goals on this topic written in for all the goals. Even with these intentions some argue that progress shows coherent implementation of actions towards the SDGs is still not occurring [81]. The separation and identification of problem areas is important, but it seems it is an easier task. Effective implementation of solutions, on the other hand, is far harder. From what we know about health inequality, linear, siloed approaches to solutions will not work. More than this, a reified understanding of interactions between and among goals is not sufficient for effective action [82]. Rather, action needs to happen on real systems focused on real interactions in the context of system behaviour—real human systems that exhibit emergence, feedbacks and sensitivity to initial conditions.

Action on the SDGs needs to consider the processes leading to large-scale inequality, which point to the need to act from an intensely local perspective. From the centrality of local lived context and agency, where voice and decision-making are currently undervalued, to its multi-level and systemic causes—where disadvantage compounds, power accumulates, and social and political polarisation is accelerating. Even in NZ where there is aspiration and stated commitment through our Voluntary National Review [83] (which showcases many of our domestic health and social policies) to contribute to achieving the SDGs, we are still finding it difficult to make a dent in our own national levels of inequality. Without achieving a real, tangible and reciprocal connection between local communities and high-level goals, poor locally embedded social and environmental outcomes will not change—and will likely worsen. The risk for how we approach action on the SDGs is that it follows the well-worn path of effort and resources accumulating at the top end of problem definition, whilst power relationships are still not devolved and little benefit trickles back down to where outcomes emerge.

## 6. Conclusions

The evidence tells us that the macro pattern of inequality is not fixed in our genes, nor is it a necessary result of human culture. Any argument that implicates genes in differences in outcomes by social group erroneously must paint a picture where there is some neutral state for social systems. There is no such neutral state, there is only underlying system behaviour, which influences the trajectory of human history, interaction, norms and culture. A big mistake of the theory behind the “invisible hand” of free-market capitalism is the belief that social systems are ever neutral, and that activities of the market, left to themselves will naturally find balance. We know this is not true, and we are seeing, the albeit unintentional, outcomes in the form of accelerating inequality and environmental degradation.

Inequality arises because of human individual and collective actions relating to group belonging and exclusion that occur over time. Cultural norms may amplify these biases, but cultural mores and values are modifiable [84]. Because of legibility and our capacity to intervene, culture should be viewed as a necessary ally. This knowledge is already widely used to influence social discord, the results of elections and the consumption of harmful products. Using it to protect human health and Earth seem more worthy goals. But intervening in culture, norms, practices and value systems will not work in a vacuum where power relationships are left undisrupted. Intentions to reduce inequality will fail without conscious and difficult actions being taken to stop the intolerable accumulation of resources; make more permeable and reciprocal the boundaries between social groups; and reverse the system flow which is currently ebbing away from the local and those already disadvantaged. Action on high-level goals such as the SDGs needs to become real and impactful and take a “whole systems” perspective on the levers it focuses on for systems change. There is substantial thinking, evidence and increasingly useful tools and concepts that can guide social action on systems change—let us begin to use them. Innovation should not be only about creating something new within the confines of existing social systems, we need innovation to transform the systems themselves.

One final note, as I am writing the revision of this article and find myself in isolation at home with my family, as NZ has gone into lockdown to try and halt the spread of Covid-19. Given the trajectories evident in our underlying systems, including the way humans are encroaching on habitats and the environment, it is clear we will experience more pandemics which may carry even greater risks. In NZ we are fortunate to currently have a low number of Covid-19 cases and a government that values scientific evidence and is therefore willing to act early. We can do this because we have good leadership, information and resources. We also have strategies in place specific to those in challenging socioeconomic circumstances, and to our Māori population. Even with these privileges and specific measures, it is likely the health and social consequences of this pandemic will fall unfairly on those already most disadvantaged. Almost certainly, this will be the stark pattern of health outcomes that emerges globally. With hope, lessons can be learned from the unprecedented social disruption the pandemic has caused. Providing a glimpse of what transformational action needs to look like for progress towards making our social and economic systems operate with more compassion, reciprocity, and intimacy within the boundaries of Earth.
